# A 3D pseudo-continuous arterial spin labeling study of altered cerebral blood flow correlation networks in mild cognitive impairment and Alzheimer's disease

**DOI:** 10.3389/fnagi.2024.1345251

**Published:** 2024-04-24

**Authors:** Meng Li, Tianjia Zhu, Yan Kang, Shouliang Qi

**Affiliations:** ^1^College of Medicine and Biological Information Engineering, Northeastern University, Shenyang, China; ^2^Department of Neurology, University of Pennsylvania, Philadelphia, PA, United States; ^3^Department of Radiology, Children's Hospital of Philadelphia, Philadelphia, PA, United States; ^4^Department of Bioengineering, School of Engineering and Applied Science, University of Pennsylvania, Philadelphia, PA, United States; ^5^College of Health Science and Environmental Engineering, Shenzhen Technology University, Shenzhen, China; ^6^Key Laboratory of Intelligent Computing in Medical Image, Ministry of Education, Northeastern University, Shenyang, China

**Keywords:** arterial spin labeling, cerebral blood flow, brain connectivity, mild cognitive impairment, Alzheimer's disease

## Abstract

**Objective:**

To investigate the abnormalities of the three-dimensional pseudo-continuous arterial spin labeling (3D PCASL) based cerebral blood flow (CBF) correlation networks in mild cognitive impairment (MCI) and Alzheimer's disease (AD).

**Methods:**

3D PCASL images of 53 cognitive normal (CN) subjects, 43 subjects with MCI, and 30 subjects with AD were acquired from the Alzheimer's Disease Neuroimaging Initiative (ADNI) database. Whole-brain CBF maps were calculated using PCASL and proton density-weighted images (PDWI). The 246 regional CBF values, including the cortex and subcortex, were obtained after registering the Brainnetome Atlas to the individual CBF maps. The Pearson correlation coefficient between every two regions across subjects was calculated to construct the CBF correlation network. Then the topologies of CBF networks with regard to global properties (global network efficiency, clustering coefficient, characteristic path length, and small-world properties), hub regions, nodal properties (betweenness centrality, BC), and connections were compared among CN, MCI, and AD. Significant changes in the global and nodal properties were observed in the permutation tests, and connections with significant differences survived after the *z*-statistic and false discovery rate (FDR) correction.

**Results:**

The CBF correlation networks of CN, MCI, and AD all showed small-world properties. Compared with CN, global efficiency decreased significantly in AD. Significant differences in nodal properties and a loss of hub regions are noted in the middle temporal lobe in both MCI and AD. In the frontal lobe, BC is reduced in MCI while it is increased in the occipital lobe in AD. The identified altered hub regions with significant differences in MCI and AD were mainly distributed in the hippocampus and entorhinal cortex. In addition, disrupted hub regions in AD with significantly decreased connections were mainly found in the precuneus/posterior cingulate cortex (PCC) and hippocampus-cortical cortex.

**Conclusions:**

Noninvasive 3D PCASL-based CBF correlation networks are capable of showing changes in topological organization in subjects with MCI and AD, and the observed disruption in the topological organization may underlie cognitive decline in MCI and AD.

## 1 Introduction

Alzheimer's disease (AD), mainly accompanied by progressive, irreversible cognitive decline, is considered the main cause of dementia (Scheltens et al., 2021). Mild cognitive impairment (MCI) is the intermediate state between normal aging and AD, mainly accompanied by memory impairment (Anderson, 2019). It is reported that AD is a disconnection syndrome (Delbeuck et al., 2003), and abnormal brain networks in MCI and AD have been proven to be associated with cognitive decline (Celone et al., 2006; Yao et al., 2010), indicating that the application of network-based methods is of significance for understanding the mechanisms of MCI and AD.

The perfusion-based functional network plays an important role in characterizing the synchronous changes of perfusion in different regions and promotes signal transmission between regions (Melie-Garcia et al., 2013; Zhu et al., 2013). The cerebral blood flow (CBF) based network is commonly constructed for functional connectivity by providing associations between non-independent regions and their properties of interregional covariation, which can be obtained by calculating the Pearson correlation coefficient (Melie-Garcia et al., 2013). It has been proven that CBF correlation networks are related to metabolic and vascular information (Luciw et al., 2021). Importantly, group-level correlation analysis is necessary for CBF networks and the stable relationship of inter-regional CBF across subjects can be captured (Melie-Garcia et al., 2013; Zhu et al., 2015). A previous study demonstrated that the episodic memory decline in MCI is associated with the alteration of the global modularity in CBF-based networks constructed with single-photon emission computed tomography (SPECT) data (Sanchez-Catasus et al., 2018). However, there are some limitations in SPECT, such as the non-repeatable examinations caused by invasive radioactive tracers, low spatial resolution, and high time consumption.

To avoid that, arterial spin-labeling (ASL) magnetic resonance imaging (MRI), with the advantages of rapid imaging and lower costs, could be applied as a strategy for measuring arterial blood flow as an endogenous tracer to assess tissue perfusion and vitality (Detre et al., 1994). Much evidence has proved that ASL MRI has a strong correlation with the functional changes related to AD and some neurodegenerative diseases (Alsop et al., 2010; Wolk and Detre, 2012). Several studies have shown that the progression of MCI and AD can be estimated by the regional CBF values with a three-dimensional pseudo-continuous ASL (3D PCASL) (Binnewijzend et al., 2013, 2016; Suzuki et al., 2023), a widely recognized technique with high labeling efficiency, repeatability, and temporal and spatial signal-to-noise ratio (Alsop et al., 2015; Dolui et al., 2017), whereas the topological properties of CBF-based networks among CN, MCI, and AD have not been estimated in earlier studies. To follow the progression of MCI and AD, we applied the 3D PCASL to detect the disrupted CBF correlation networks in MCI and AD. Graph theory was used to characterize the topologies by exploring the regional and global properties, and significant differences (p < 0.05) were obtained by comparing the results between each two groups. Finally, a *z*-statistic was calculated to obtain the significantly altered connections with the false discovery rate (FDR) correction.

## 2 Materials and methods

### 2.1 3D PCASL data from ADNI

The 3D PCASL data for this study were acquired from the Alzheimer's Disease Neuroimaging Initiative (ADNI) database (adni.loni.usc.edu). ADNI was launched in 2003 as a public–private partnership, led by Principal Investigator Michael W. Weiner, MD. The primary goal of ADNI has been to test whether serial MRI, positron emission tomography (PET), other biological markers, and clinical and neuropsychological assessments can be combined to measure the progression of MCI and early AD. For up-to-date information (see www.adni-info.org).

Each subject is assigned a unique identification number. CN subjects have a Memory Box score of 0, a Clinical Dementia Rating (CDR) of 0, and a Mini-Mental Status Exam (MMSE) score between 24 and 30. Participants with memory loss and who have a Memory Box score of at least 0.5, a CDR score of 0.5, and an MMSE score between 24 and 30 are diagnosed as having MCI. Subjects diagnosed with AD have a CDR of 0.5 or 1.0 and a MMSE score between 20 and 24 following the National Institute of Neurological and Communicative Disorders and Stroke-Alzheimer's Disease and Related Disorders Association (NINCDS/ADRDA). Detailed inclusion and exclusion criteria can be found at https://adni.loni.usc.edu/wp-content/themes/freshnews-dev-v2/documents/clinical/.

### 2.2 Clinical assessment

All subjects from ADNI completed a comprehensive neuropsychological assessment based on standardized tests. Measures associated with cognitive domains include MMSE, Montreal Cognitive Assessment (MoCA), and Alzheimer's Disease Assessment Scale-Cognitive (ADAS13). The demographic and clinical information was statistically analyzed by using MATLAB, one-way analysis of variance (ANOVA), and *post hoc* tests were applied to compare variables (age, education level, MMSE, MoCA, and ADAS13) among three groups. Chi-squared test was used to determine if there is any significant relationship between sex (male/female) and subjects with CN, MCI, and AD.

### 2.3 Image acquisition

Both high-resolution structural MRI data and resting PCASL data were downloaded for 53 CN subjects, 43 MCI subjects, and 30 AD subjects. The structural images were acquired using an accelerated sagittal inversion recovery fast spoiled gradient recall (IR-FSPGR) T1-weighted sequence with the following parameters: repetition time (TR)/echo time (TE)/inversion time (TI) = 7.39/3.06/400 ms, 196 sagittal slices, slice thickness = 1.0 mm, within the plane field of view (FOV) =256 × 256 × 196 mm^3^, voxel size = 1 × 1 × 1 mm^3^.

3D PCASL data (label/control images) were acquired by a 3.0 T MRI scanner (Discovery MR 750, GE Medical Systems) with background suppression and no vascular suppression. The acquisition parameters were as follows: TR/TE = 4,888/10.5 ms, TI = 2,025 ms, post labeling delay (PLD) = 2,000 ms, slice thickness = 4.0 mm, FOV = 24 × 24 cm^2^, weighting = proton density (PD).

### 2.4 Overview of CBF correlation network analysis

**Figure 1:**
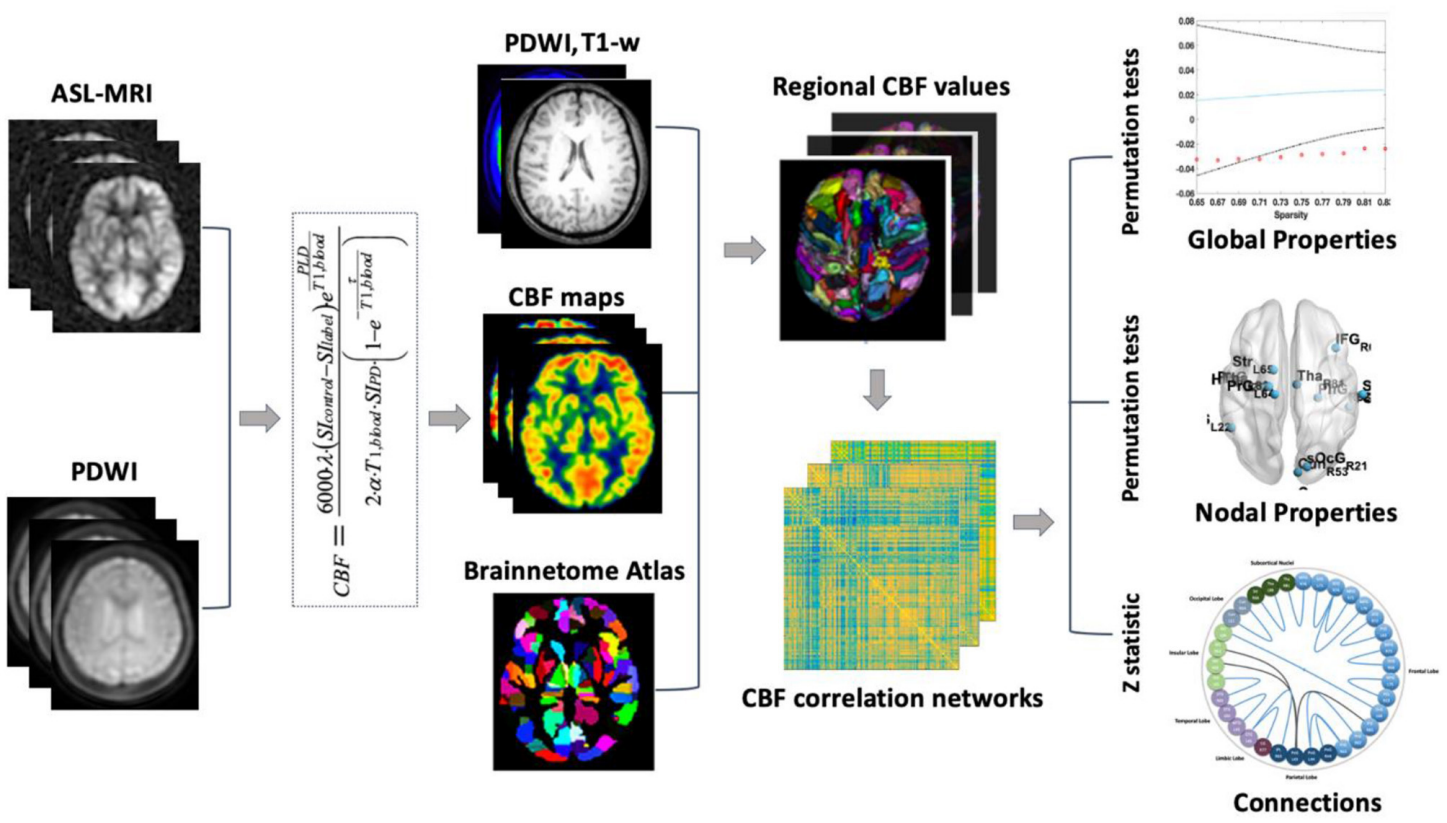
Study procedure of CBF correlation network analysis.

The processing steps of CBF correlation network analysis by using the 3D PCASL MRI are shown in Figure 1. First, the 3D PCASL MRI and PDWI were applied to calculate the CBF map (see “2.5 CBF maps Calculation”). Second, the Brainnetome Atlas (Fan et al., 2016) was registered from MNI space to individual space with T1 and proton density-weighted images (PDWI). Thus the CBF map was divided into 246 regions in gray matter. Third, the Pearson coefficient between regional CBF values at the group level was calculated for the construction of the correlation networks (see “2.6 Network Construction”). Finally, the changes in global properties (see “2.7 Global Properties Analysis”), hub regions (see “2.8 Hub regions in CBF correlation networks”), and nodal property (see “2.9 Nodal Property Analysis”), as well as altered connections were analyzed (see “2.10 Connection Analysis”).

### 2.5 Calculation of CBF maps

The data of 3D PCASL (label/control images) and PDWI were converted into the 4D.nii.gz in NIFTI from DICOM format by using the tools of dcm2nii (https://www.nitrc.org/projects/dcm2nii). Then they were processed by FSL (https://fsl.fmrib.ox.ac.uk/fsl/fslwiki/) with motion correction and brain tissue extraction to benefit the following processing and analysis.


$$\begin{array}{l} CBF=\frac{6000\cdot \lambda \cdot \left({SI}_{control}-{SI}_{label}\right)\cdot e^{\frac{PLD}{T1,blood}}}{2\cdot \alpha \cdot T_{1,blood}\cdot {SI}_{PD}\cdot \left(1-e^{-\frac{\tau }{T1,blood}}\right)} \end{array}$$

The above formula was applied to calculate CBF in the whole brain. The scan parameters are as follows: *T*1 of blood is assumed to be 1.4 s, the factor τ represents the label duration which is 1.5 s, λ is the brain/blood partition coefficient whose value is 0.9 (ml/g), PLD is the time of post-labeling delay and is set to 2.025 s, α is the labeling efficiency factor with a value of 0.8.

### 2.6 Network construction

Gray matter brain regions were obtained from CBF maps by using the FSL tools. Each subject's PDWI image was first registered to T1 data linearly, and a matrix with mutual information was obtained, with linear and non-linear methods. We normalized T1 data to MNI space. Next, the co-registered PDWI image in structure space was warped by the transform field normalized from T1 to MNI. Then, the Brainnetome Atlas in MNI space was registered to individual PCASL and PDWI space through the results we obtained in the last step (Yuan et al., 2017). Thus 246 regions in each subject were obtained with the individual atlas and whole brain CBF maps.

To construct the CBF connectivity network, three CBF data matrices for CN (53 × 246), MCI (43 × 246), and AD (30 × 246) were prepared, in which rows represent the number of subjects and columns represent the number of brain regions, and Pearson's correlation index between each two brain regions across subjects was calculated and three CBF connectivity networks were constructed. Then the CBF connectivity networks after normalization were calculated at different sparsity from 0.65 to 0.83 with the step size 0.02.

### 2.7 Analysis of global properties

To analyze the global properties of the CBF connectivity network, we calculated the global network efficiency, clustering coefficient, characteristic path length, and small-world properties. The global efficiency is the average inverse shortest path length in the network and is inversely related to the characteristic path length. The clustering coefficient is the fraction of triangles around a node and is equivalent to the fraction of the node's neighbors that are neighbors of each other. The small-world properties are related to the normalized average clustering coefficient and the average shortest path length. When the small-world coefficient value is >1, we consider it to have the small-world property.

We applied the non-parametric permutation approach (1,000 permutations) to compare the global network properties between every two groups. In each permutation, regional CBF values were randomly reassigned and the number of subjects in each randomized group was the same as that in the original group with CN (53 × 246), MCI (43 × 246), and AD (30 × 246), and the correlation connectivity network was constructed for each randomized group. We obtained the global parameters of randomized matrices at a range of sparsity 0.65–0.83 with step size 0.02 in each group with correlation connectivity networks constructed by each randomized group. Finally, the difference in the global network parameters of original networks was compared to that of randomized networks (null distributions) and the relative positions of difference as nonparametric *p*-values were obtained, in which parameters with *p*-values under 0.05 were considered to be significant.

### 2.8 Hub regions in CBF correlation networks

The MATLAB-based Brain Connectivity Toolbox (BCT) for complex brain network analysis (Rubinov and Sporns, 2010) was applied to analyze the properties of the CBF connectivity network in CN, MCI, and AD groups.

We identified the hub regions with node measure betweenness centrality (BC) and the fraction of all shortest network paths containing a given node, which is applied most widely in characterizing the importance of nodes in a network (Freeman, 1977). In the current study, nodes with BC two times higher than the mean value of all the nodes were defined as hub regions. Hub regions in the CN group were first detected, and then the corresponding BC values of regions in MCI and AD groups were calculated.

### 2.9 Nodal property analysis

To detect the changes in nodal properties of the CBF connectivity network among CN, MCI, and AD groups, the non-parametric permutation approach (1,000 permutations) was applied. With the same method described in the global properties analysis, we constructed the original and randomized correlation networks, and then BC values of nodes in original and randomized matrices at a sparsity of 0.83 in each group were obtained. Finally, the difference of BC values of nodes in the original networks was compared to that of randomized networks (null distributions), and the relative positions of difference as non-parametric *p*-values were obtained, nodes with significant differences were identified with *p*-values below 0.05.

### 2.10 Analysis of connections

Significant differences in the connections of CBF connectivity networks with a sparsity of 0.83 between every two groups (CN vs. MCI, CN vs. AD, and MCI vs. AD) were tested. z-values were obtained from the correlation coefficients with Fisher's *z*-transformation. Then a *z*-statistic was obtained, and connections with significantly different values survived with false discovery rate (FDR) correction (*p* < 0.001).

## 3 Results

### 3.1 Demographic and clinical characteristics of ADNI subjects

The demographic and clinical characteristics of subjects from ADNI are shown in Table 1. Significant differences (p < 0.001) were found among CN, MCI, and AD in MMSE, MoCA, and ADAS13. No significant difference was found among the three groups in terms of age, sex, and education level.

**Table 1:** Demographic and clinical characteristics of ADNI subjects.

**Parameter**	**CN**	**MCI**	**AD**	***P* value**
	**(*****n*** = **53)**	**(*****n*** = **43)**	**(*****n*** = **30)**	
Age, years	74.1 ± 3.2	76.2 ± 3.6	75.1 ± 5.3	0.243^a^
Education, years	16.3 ± 2.1	16.4 ± 2.3	16.6 ± 2.2	0.537^a^
Sex, menwomen/men	26/27	22/21	16/14	0.931^b^
MMSE	28.9 ± 2.4	26.8 ± 3.4	21.5 ± 2.7	< 0.001^a*^
MoCA	25.7 ± 2.6	23.1 ± 3.9	17.6 ± 3.9	< 0.001^a*^
ADAS13	7.9 ± 4.5	16.9 ± 4.1	27.8 ± 2.9	< 0.001^a*^

Data are shown as the mean ± standard deviation. ^*^P < 0.05; ^a^one-way analysis of variance; ^b^chi-squared test; ADNI, Alzheimer's Disease Neuroimaging Initiative; CN, cognitive normal; MCI, Mild cognitive impairment; AD, Alzheimer's disease; MMSE, Mini-Mental Status Exam; MoCA, Montreal Cognitive Assessment; ADAS13, Alzheimer's Disease Assessment Scale-Cognitive13.

### 3.2 Calculated CBF maps and regional CBF values

**Figure 2:**
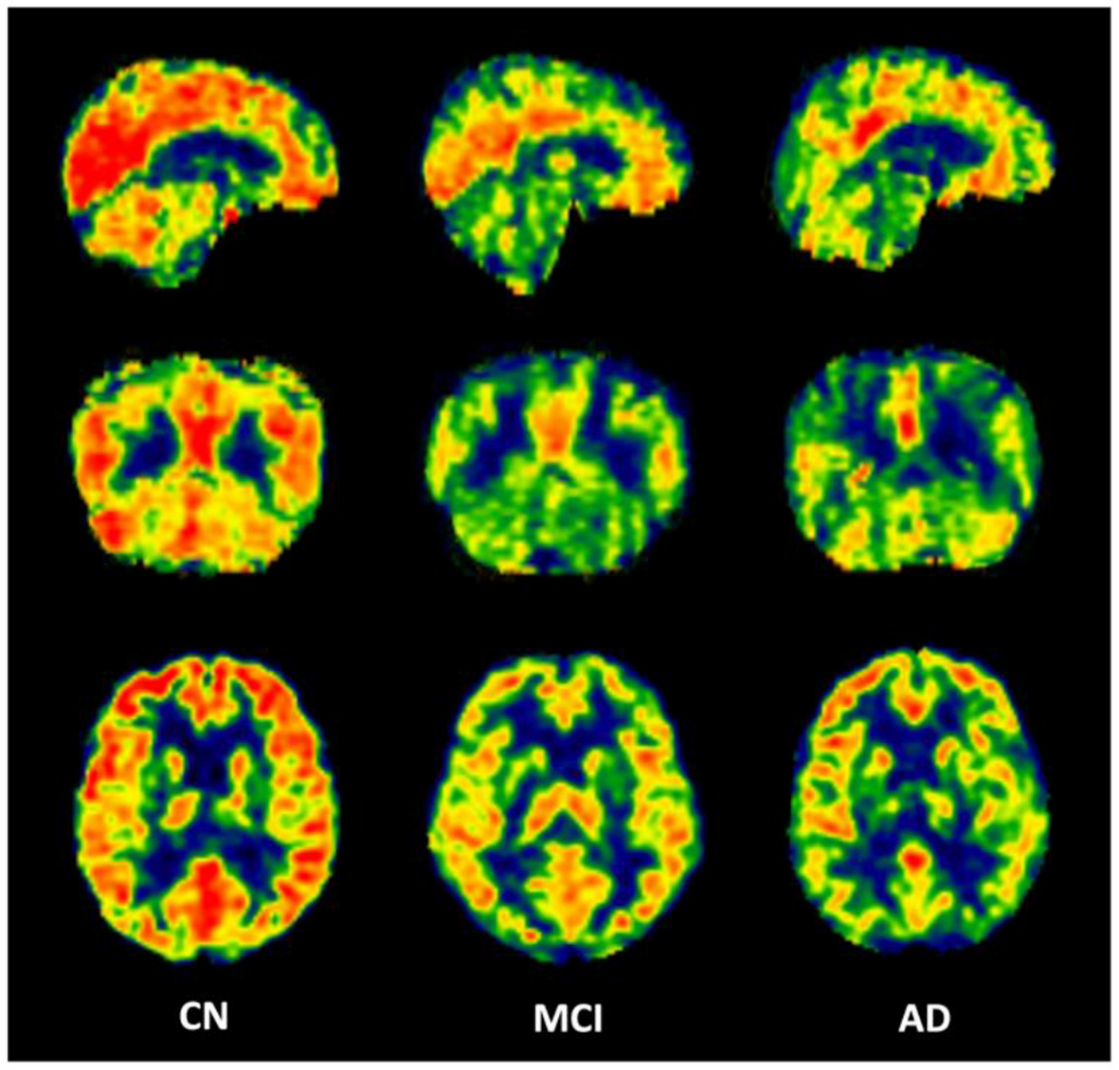
Representative CBF maps from CN, MCI, and AD groups.

**Figure 3:**
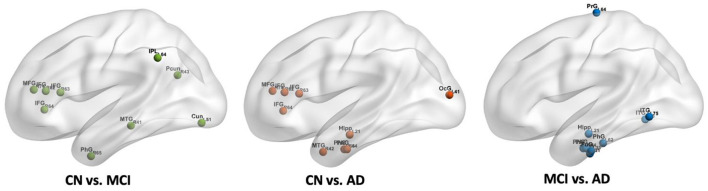
The spatial locations of regions with significant differences in CBF values among CN, MCI, and AD groups.

The calculated whole brain CBF maps of CN, MCI, and AD are shown in Figure 2. After the registration of the Brainnetome Atlas to CBF maps in individual space, 246 regional CBF values in gray matter were obtained. Regional CBF with significant differences among CN, MCI, and AD were statistically analyzed with double t-tests and FDR correction (*p* < 0.05). Compared with CN, nine regions decreased significantly in MCI, mainly distributed in the frontal gyrus, middle temporal gyrus, parahippocampal gyrus, parietal lobe, and occipital lobe. Compared with CN and MCI, nine regions with significant differences were found in AD, they are mainly in the frontal gyrus, temporal gyrus, parahippocampal gyrus (e.g., entorhinal cortex), and hippocampus. Figure 3 shows the spatial location of the regions mentioned above, and the regional values with significant differences are listed in Supplementary Table 1.

### 3.3 Global properties of CBF correlation networks

**Figure 4:**
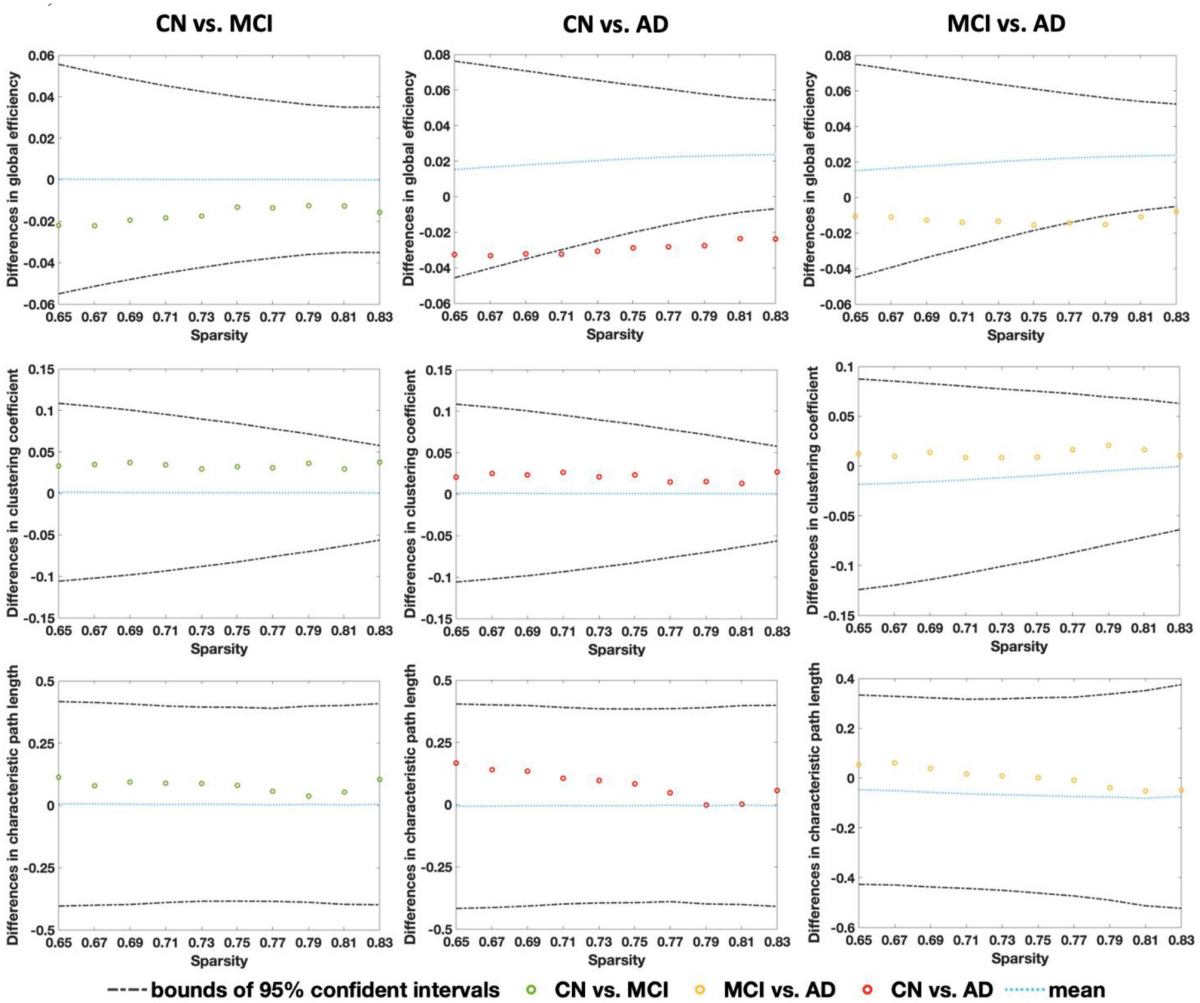
Relative differences in global properties of CBF correlation networks (global efficiency, clustering coefficient, and characteristic path length) between CN-MCI, CN-AD, and MCI-AD across the sparsity from 0.65 to 0.83. The non-parametric permutation tests (1,000 permutations) were conducted showing the results of the mean value (blue lines) and 95% confidence intervals (dashed lines).

**Figure 5:**
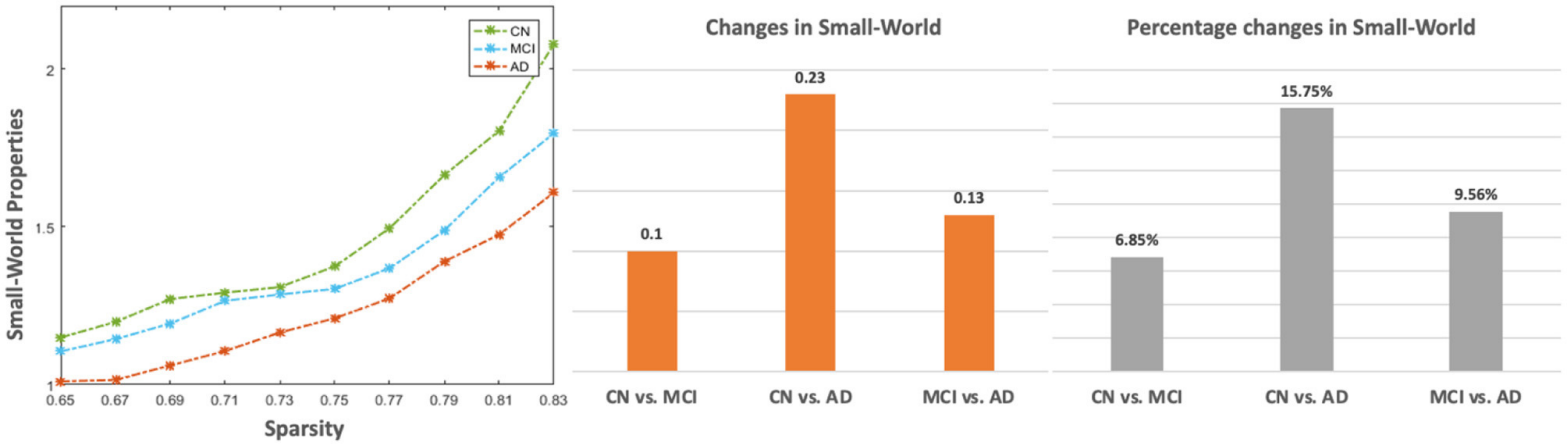
The small-world properties of the CBF correlation network in CN, MCI, and AD groups. The left sub-figure is the small-world values across the sparsity from 0.65 to 0.83. The two sub-figures on the right are absolute and percentage changes in mean small-world by averaging across all sparsity levels among CN, MCI, and AD, respectively.

From Figure 4, global efficiency shows a significant decrease in the AD group compared with the CN group under the sparsity of 0.71 (*p* = 0.031), 0.73 (*p* = 0.016), 0.75 (*p* = 0.009), 0.77 (*p* = 0.002), 0.79 (*p* = 0.002), 0.81 (*p* < 0.001), and 0.83 (*p* < 0.001). A significant decrease was also found from a comparison between MCI and AD at the sparsity of 0.77 (*p* = 0.027), 0.79 (*p* = 0.018), 0.81 (*p* = 0.003). No significant changes were found among the CN, MCI, and AD for clustering coefficient and characteristic path length. Figure 5 shows that all three groups exhibit small-world topologies across the sparsity from 0.65 to 0.83, and the intermediate state of MCI is shown between CN and AD in the small-world properties across all the sparsity values. From the bar plot in Figure 5, we found that, compared with CN, the mean small-world value of all sparsity in MCI decreased by 0.1 (6.85%), and that in AD decreased by 0.23 (15.75%). Meanwhile, the mean small-world value in AD decreased by 0.13 (9.56%) compared with MCI.

### 3.4 Hub regions in CBF correlation networks

**Figure 6:**
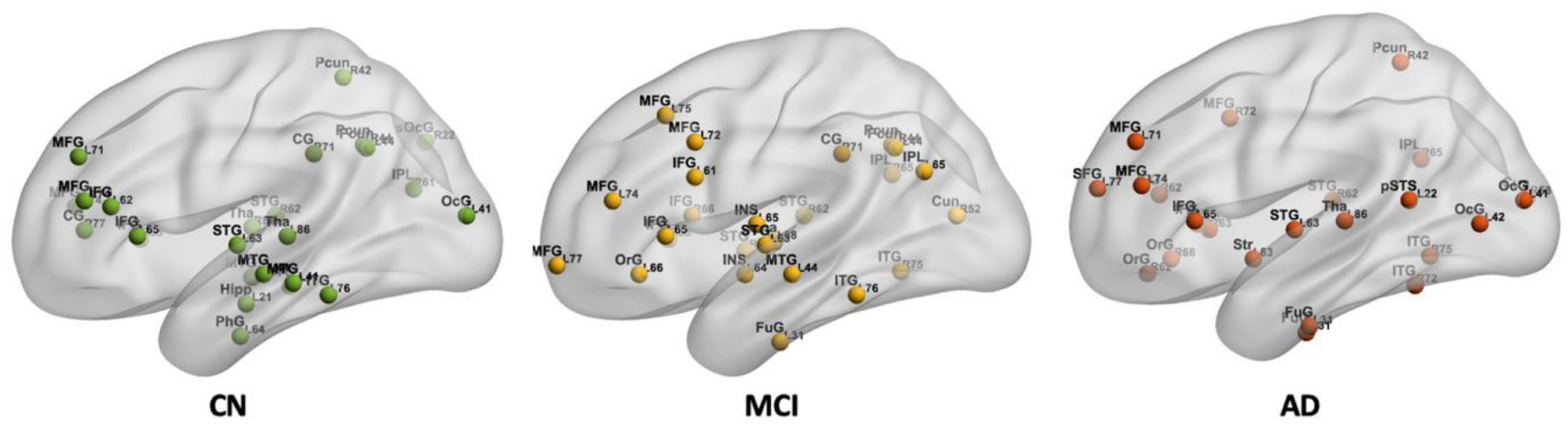
The spatial locations of hub regions in CN, MCI, and AD.

Hub regions in the CN, MCI, and AD are shown in Figure 6. Twenty-five hub regions were identified in CN, while the number of hub regions is 25 in MCI and 24 in AD. Hub regions were identified in three groups distributed in the inferior and middle frontal gyrus, orbital gyrus, inferior and superior temporal gyrus, precuneus, and occipital gyrus. The identified hub regions are listed in Supplementary Table 2.

In addition, the middle temporal gyrus, inferior parietal lobule, and cingulate gyrus including cingulate gyrus (CG)_R_7_1 (i.e., posterior cingulate cortex, PCC) were found as hub regions in CN and MCI but disappeared in AD. Identical hub regions such as parahippocampal gyrus (PhG)_L_6_4 (i.e., entorhinal cortex), and hippocampus (Hipp)_L_2_1 were also found only in CN. For both MCI and AD groups, hub regions were found in the insular gyrus and cuneus. Additionally, hub regions in the striatum and posterior superior temporal sulcus were also observed.

### 3.5 Altered nodal property in CBF correlation networks

**Figure 7:**
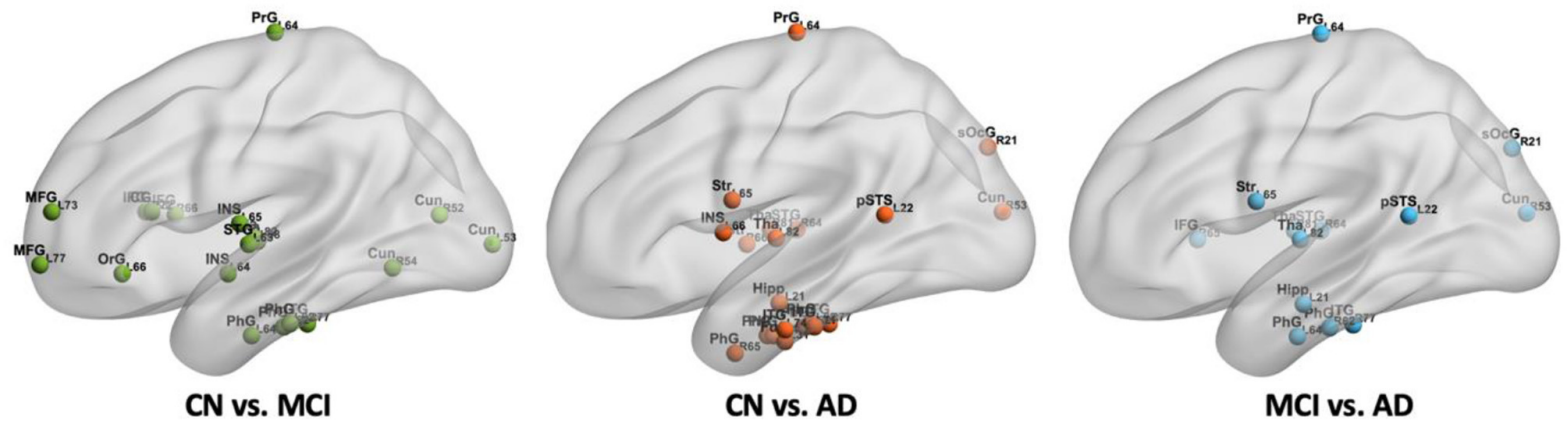
The spatial locations of regions with significant differences in nodal BC value in CBF correlation networks among CN, MCI, and AD groups.

From Figure 7, we found that 18 regions with significantly different BC values in the CBF correlation networks between CN and MCI groups, including the inferior frontal gyrus, middle frontal gyrus, dorsal anterior insula, inferior temporal gyrus, orbital gyrus, cuneus, parahippocampal gyrus, superior temporal gyrus, and thalamus. Between CN and AD groups, 20 regions were detected with significant differences. They were distributed in the parahippocampal gyrus, cuneus, caudoventral anterior insula, inferior temporal gyrus, superior temporal gyrus, striatum, thalamus, fusiform gyrus, hippocampus, posterior superior temporal sulcus, and superior occipital gyrus. Compared with the MCI group, 13 regions were found to be decreased in the AD group, which include the cuneus, hippocampus, inferior frontal gyrus, inferior temporal gyrus, parahippocampal gyrus, posterior superior temporal sulcus, superior occipital gyrus, superior temporal gyrus, striatum, thalamus, and precentral gyrus. In addition, we found cuneus, inferior temporal gyrus, parahippocampal gyrus, superior temporal gyrus, and thalamus changed significantly between every two groups. Importantly, the significantly changed BC value of PhG_L_6_4 (i.e., entorhinal cortex) was found in MCI and AD. We found that there is no region with significant differences between CN and MCI in the hippocampus, and with progression and the BC value of Hipp_L_2_1 in the hippocampus decreased significantly in AD. We also found that with progression, significantly different regions in the frontal lobe decreased, while some regions in the occipital lobe increased significantly in AD. The regions with significantly different BC values are listed in Supplementary Table 3.

### 3.6 Altered connections in CBF correlation networks

**Figure 8:**
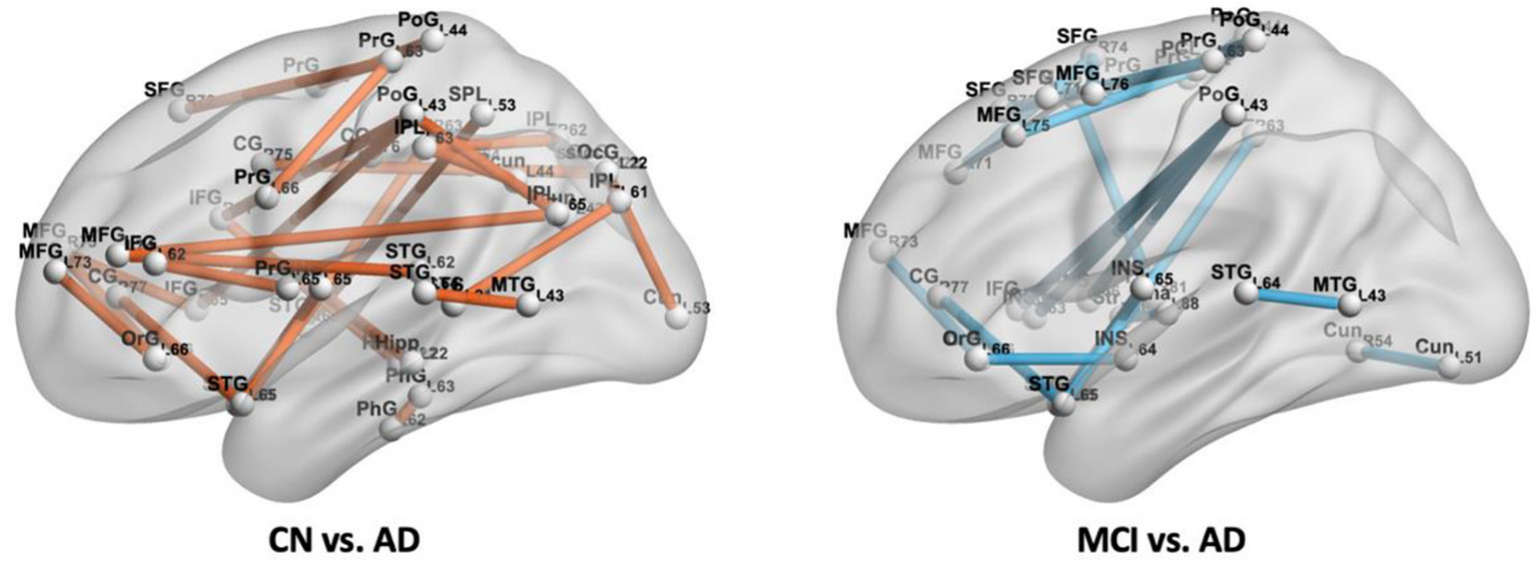
Connections with significant difference (*P* < 0.001) between CN and AD, MCI and AD.

**Figure 9:**
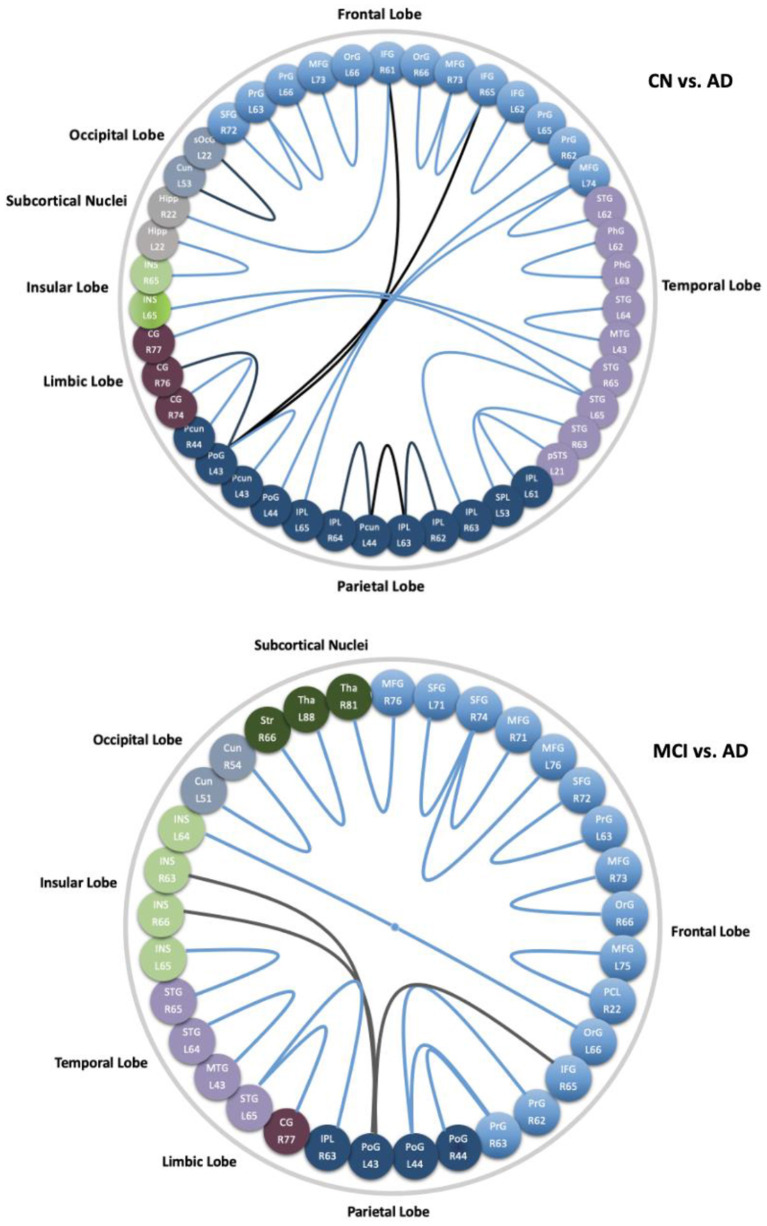
Subregions and lobes to which the corresponding connections with significant differences of CN vs. AD (**top**), and MCI vs. AD (**bottom**). Connections in blue decrease significantly in AD, while the black ones are increased edges in AD.

We did not find a connection with significant differences between CN and MCI. While 27 connections were found with significant differences between CN and AD, they were distributed in the frontal lobe (middle and inferior frontal gyrus, orbital gyrus, precentral gyrus), temporal lobe (superior and middle temporal gyrus, parahippocampal gyrus, posterior superior temporal sulcus), parietal lobe (superior parietal lobule, angular gyrus, supramarginal gyrus, precuneus, postcentral gyrus), insular lobe (rostrodorsal posterior insula), limbic lobe (cingulate gyrus), occipital lobe (cuneus, superior occipital gyrus), and subcortical nuclei (hippocampus), and those spatial distributions mentioned above are shown in Figure 8 (left). From Figure 9 (top), we found most of the connections were significantly decreased, except for seven connections in the frontal, parietal limbic, and occipital lobes were increased. Compared with MCI, we found 20 connections with significant differences in the AD group, which were distributed in the frontal lobe (superior, middle and inferior frontal gyrus, orbital gyrus, precentral gyrus, paracentral lobule), temporal lobe (superior and middle temporal gyrus), parietal lobe (supramarginal gyrus, postcentral gyrus), insular lobe (dorsal anterior insula, ventral posterior insula, rostrodorsal posterior insula, caudoventral anterior insula), limbic lobe (cingulate gyrus), occipital lobe (cuneus), and subcortical nuclei (striatum, thalamus). The spatial distributions of these connections are shown in Figure 8 (right). From Figure 9 (bottom), three edges in the frontal, parietal, and insular lobe increased significantly, and the other edges reduced significantly in AD compared with MCI.

## 4 Discussion

We used a 3D PCASL technique to investigate the altered topological properties of CBF correlation networks in subjects identified as having CN, MCI, and AD. In earlier studies, correlation networks have been detected with different techniques for MCI or AD, including functional networks with SPECT data, fluorodeoxyglucose positron emission tomography (FDG-PET) data, blood oxygen level-dependent (BOLD) MRI, and cortical networks with T1-weighted MRI (Yao et al., 2010; Seo et al., 2013; Dai and He, 2014; Sanchez-Catasus et al., 2018). However, CBF correlation networks were only analyzed in MCI with SPECT (Sanchez-Catasus et al., 2018), where the global efficiency of MCI was found to be reduced compared to the control group. Although FDG-PET, SPECT, and ASL are widely applied to measure cerebral metabolism, ASL has the advantages of rapid imaging, non-invasive, and low costs compared with the others. BOLD and ASL are both commonly used to estimate functional networks calculated by interregional correlation coefficients. Using BOLD, time series were used to examine the connectivity at the individual level, which can reflect temporal synchronization of the inter-regional neural activity (Biswal et al., 1995), while the interregional CBF-related networks are obtained at the group level and cannot be compared between individuals. Moreover, CBF connectivity as a single physiological parameter might be more relevant for characterizing cerebral metabolism, whereas BOLD connectivity is influenced by several parameters including CBF, cerebral blood volume, cerebral metabolic rate of oxygen, and oxygen metabolism (Buxton et al., 2004).

There are five main findings revealed in this study: (1). The CBF correlation networks of CN, MCI, and AD showed small-world properties. (2). Compared with CN, global efficiency showed a significant decrease in AD. (3). A loss of hub regions in the middle temporal lobe was found in MCI and AD. (4). Significant differences in the nodal properties of BC are observed in both MCI and AD in the middle temporal lobe; in addition, in the frontal lobe, BC was found to be reduced in MCI while it increased in the occipital lobe in AD. Both regional CBF values and BC decreased in the entorhinal cortex and hippocampus in AD. (5). Connections in AD showed significant differences compared to those in CN and MCI. The findings mentioned above are discussed in the following subsections.

### 4.1 Global properties of CBF correlation networks

Compared with matched random networks, small-world networks have higher local clustering and similar path lengths, thus improving the communication efficiency of the brain (Bullmore and Sporns, 2009). In this study, the CN, MCI, and AD groups all exhibited small-world properties, which are consistent with other studies including FDG-PET-based functional and cortical networks in MCI and AD (Supekar et al., 2008; Yao et al., 2010; Seo et al., 2013; Xu et al., 2020), indicating that the 3D PCASL-based CBF correlation networks of CN, MCI, and AD present an optimal balance between local specialization and global integration processes.

In comparison with CN and MCI, the global efficiency was reduced in AD, which is consistent with a prior study (Liu et al., 2014), where the lower global efficiency of BOLD functional MRI networks in AD was reported. While in other studies, significantly reduced global efficiency or the clustering coefficient of networks were found (Berlot et al., 2016; Sanchez-Catasus et al., 2018; Zhou, 2020), in this study, MCI shows no apparent differences in properties of global efficiency, clustering coefficient, and characteristic path length over the entire range of sparsity thresholds. The intermediate state of MCI of the small-world in CBF correlation networks between CN and AD is consistent with prior studies (Liu et al., 2012; Dai and He, 2014).

### 4.2 Hub regions in CBF correlation networks

The hub regions identified in our study are largely consistent with previous reports. For instance, in CN, hub regions in the middle temporal gyrus, inferior frontal gyrus, and orbital gyrus occurred in a previous study in which cortical networks were characterized by gray matter volumes (Yao et al., 2010). Hub regions in the precuneus, medial frontal gyrus, inferior parietal cortex, and thalamus in current findings are consistent with the results in a study where nodal strength was used to identify hubs in functional networks (Dai et al., 2015). For the altered hub regions in brain connectivity in MCI and AD, some of our results are consistent with previous studies. Hub regions in the right precuneus were defined in all three groups, with left parahippocampal gyrus (e.g., entorhinal cortex) only appearing in the CN but missing in MCI and AD (Seo et al., 2013; Khazaee et al., 2017). In agreement with earlier studies (Seo et al., 2013; Grajski et al., 2019; Hrybouski et al., 2023), hub regions were found in the left precuneus and PCC only in CN and MCI, while in MCI and AD, hub regions occurred in the right angular gyrus (right inferior parietal lobe). Moreover, hub regions in the middle temporal gyrus in CN disappearing in MCI an AD was also proved. Of note, the loss of hubs in the hippocampus and entorhinal cortex in MCI and AD are subregions of the core memory-associated middle temporal lobe, which might be related to the dysfunction of the memory (Fjell et al., 2015; Hrybouski et al., 2023).

### 4.3 Altered CBF values and nodal properties in CBF correlation networks

Brain regions with abnormal nodal properties (in terms of BC) and regional CBF values in AD are mainly distributed in the parahippocampal gyrus (e.g., entorhinal cortex), temporal gyrus, and hippocampus. The regions with significant differences in the entorhinal cortex and hippocampus are also identified as the loss hub regions, which is consistent with prior research and related to the impairment of memory (Seo et al., 2013; Hrybouski et al., 2023). Compared with CN, altered BC was found in the inferior frontal gyrus in MCI, which was revealed in a prior study (Seo et al., 2013). The superior temporal gyrus was found to decrease significantly in BC in MCI and AD, which was also marked in a previous study (He et al., 2008; Zhang et al., 2021), which involved auditory (e.g., language) processing and social cognition (Bigler et al., 2007). Additionally, significant BC increased in the occipital gyrus (e.g., cuneus). The increased hub region in AD, is consistent with a study in which structural connectivity was applied to understanding the association between disrupted integrity of the network and the underlying cognition (He et al., 2008), and which may serve as a compensatory system.

A previous study proved that regional CBF values decreased in patients with AD, and they were mainly distributed in the entorhinal cortex and hippocampus. The regional CBF values in the parietal cortex and precuneus were reduced in MCI when compared with CN, and our findings are consistent with these results (Binnewijzend et al., 2013; Mattsson et al., 2014). Moreover, a higher amyloid-β load was considered to have associations with those lower regional CBF values mentioned above (Maier et al., 2014; Mattsson et al., 2014). Although we did not estimate the association between the amyloid-β load and network-based changes of CBF, the abnormality of hub regions and connections in the entorhinal cortex and hippocampus in AD in this study may be related to the high load of amyloid-β.

### 4.4 Altered connections in CBF correlation networks

In Figure 9, details of the changes with significant differences (P < 0.001) of groups with CN-AD and MCI-AD are shown. Compared with CN, the decreased correlations in AD are mainly distributed in the frontal lobe, temporal lobe (e.g., middle temporal gyrus and parahippocampus), parietal lobe (e.g., precuneus and cingulate gyrus), and subcortical nuclei (e.g., hippocampus). The disrupted hub regions with apparently reduced BC are also linked by significantly decreased edges. They occurred in parahippocampus, precuneus, and hippocampus. Most of the altered connections in the hippocampus, middle temporal gyrus, cingulate gyrus, and precuneus in current CBF correlation networks are consistent with changes in the functional networks of AD in earlier studies (Wang et al., 2006; Zhou et al., 2008; Seo et al., 2013). Specifically, the alteration of the hippocampal–cortical (e.g., hippocampal-inferior frontal gyrus) connectivity is consistent with previous studies (Wang et al., 2006; Allen et al., 2007) and may link the decline of memory and cognitive function in AD since the hippocampal-cortical memory system contains interacting brain regions that are activated during episodic memory retrieval (Vincent et al., 2006; Buckner et al., 2008). In addition, the connection of the hippocampus to the right insular reduced in AD is in agreement with a prrevious study (Wang et al., 2011) and the changes in connections may underlie memory impairment. In earlier studies, the inferior parietal gyrus and/or precuneus were proved to have an association with mental orientation in CN and aMCI (Peer et al., 2015; Oishi et al., 2018). Our findings in this study showed the connection of the precuneus to the inferior parietal lobe decreased significantly, which may be the underlying reason for cognitive dysfunction of disorientation.

Moreover, the disruption of hub regions with altered connections occurred in the middle temporal gyrus, middle frontal gyrus, and cingulate gyrus. Of note, connectivity between precuneus and PCC decreased significantly in AD, which is consistent with prior studies (Rami et al., 2012; Yokoi et al., 2018). The functional connectivity with significantly decreased precuneus/PCC was assumed to have an association with cognitive function and plays a key role in developing AD (Yokoi et al., 2018).

### 4.5 Limitations

This study has several limitations. First, the modest sample size of 3D PCASL data from the ADNI database may limit its statistical power. Second, the results of the CBF map calculations were affected by the parameters selected. Third, in this study, we only studied the group-level correlation networks due to the limitations of time series with current GE ASL data, and the individual-level networks were not analyzed. Finally, we found significant differences in cognitive scores (MMSE, MoCA, and ADAS13) among the three groups, while no correlation analysis of cognitive scores and network parameters of CN, MCI, and AD was performed in this study.

## 5 Conclusion

In this study, we estimated the abnormality of the topological organization of 3D PCASL-based CBF correlation networks in subjects with MCI and AD. The CBF correlation networks of CN, MCI, and AD all showed small-world properties. Compared with CN, global efficiency decreased significantly in AD. Significant differences in nodal properties and a loss of hub regions were observed in the middle temporal lobe in both MCI and AD. In the frontal lobe, BC was reduced in MCI while it increased in the occipital lobe in AD. The identified altered hub regions with significant differences in MCI and AD were mainly distributed in the hippocampus and entorhinal cortex. In addition, disrupted hub regions in AD with significantly decreased connections were mainly found in the precuneus/PCC and hippocampus-cortical cortex. The observed disruptions in the topological organization of 3D PCASL-based CBF correlation networks may underlie the cognitive decline and provide new insight into understanding the mechanism of MCI and AD.

## Data availability statement

Publicly available datasets were analyzed in this study. This data can be found here: Alzheimer's Disease Neuroimaging Initiative (ADNI) database (www.adni-info.org).

## Ethics statement

The studies involving humans were approved by according to Good Clinical Practice guidelines, US 21Code of Federal Regulations Part 50– Protection of Human Subjects, and Part 56 –Institutional Review Boards/Research Ethics Boards, and under state and federal HIPAA regulations. The studies were conducted in accordance with the local legislation and institutional requirements. The participants provided their written informed consent to participate in this study.

## Author contributions

ML: Conceptualization, Data curation, Formal analysis, Investigation, Methodology, Software, Writing – original draft. TZ: Investigation, Writing – review & editing. YK: Conceptualization, Supervision, Writing – review & editing. SQ: Conceptualization, Funding acquisition, Supervision, Writing – review & editing.
