# Supplementary material for: A 3D pseudo-continuous arterial spin labeling study of altered cerebral blood flow correlation networks in mild cognitive impairment and Alzheimer's disease
Source: Front Aging Neurosci. 2024 Apr 24;16:1345251. doi: 10.3389/fnagi.2024.1345251 (PMC11077634; doi:10.3389/fnagi.2024.1345251)
Supplement: Supplementary file 1 [file Table_1.DOCX]

**Supplementary**

**Table 1. Regions with significant differences in CBF values among the CN, MCI, and AD**

| **CN vs. MCI** | **P value** | **CN vs. AD** | **P value** | **MCI vs. AD** | **P value** |
| --- | --- | --- | --- | --- | --- |
| Middle Frontal Gyrus_R_7_4 | 0.021 | Hippocampus_L_2_1 | 0.003 | Hippocampus_L_2_1 | 0.012 |
| Inferior Frontal Gyrus_R_6_2 | 0.022 | Middle Frontal Gyrus_R_7_4 | 0.001 | Precentral Gyrus_R_6_4 | 0.005 |
| Inferior Frontal Gyrus_R_6_3 | 0.001 | Inferior Frontal Gyrus_R_6_2 | 0.019 | Inferior Temporal Gyrus_L_7_3 | 0.004 |
| Inferior Frontal Gyrus_R_6_4 | 0.013 | Inferior Frontal Gyrus_R_6_3 | 0.002 | Inferior Temporal Gyrus_R_7_3 | 0.026 |
| Middle Temporal Gyrus_R_4_1 | 0.010 | Inferior Frontal Gyrus_R_6_4 | 0.016 | Fusiform Gyrus_L_3_1 | 0.005 |
| Parahippocampal Gyrus_R_6_5 | 0.019 | Middle Temporal Gyrus_R_4_2 | 0.037 | Fusiform Gyrus_R_3_1 | 0.006 |
| Inferior Parietal Lobule_L_6_4 | 0.032 | Parahippocampal Gyrus_L_6_4 | 0.023 | Parahippocampal Gyrus_L_6_4 | 0.001 |
| Precuneus_R_4_3 | 0.035 | Parahippocampal Gyrus_R_6_4 | 0.026 | Parahippocampal Gyrus_R_6_4 | 0.013 |
| Cuneus_L_5_1 | 0.017 | Occipital Gyrus_L_4_1 | 0.018 | Parahippocampal Gyrus_L_6_2 | 0.004 |

**Table 2. Hub regions identified in the CN, MCI, and AD**

| **CN** | **BC value** | **MCI** | **BC value** | **AD** | **BC value** |
| --- | --- | --- | --- | --- | --- |
| Middle Temporal Gyrus_R_4_4 | 10.307 | Superior Temporal Gyrus_L_6_3 | 10.706 | Inferior Frontal Gyrus_R_6_5 | 10.998 |
| Precuneus_R_4_4 | 9.862 | Precuneus_R_4_4 | 7.933 | Inferior Frontal Gyrus_L_6_5 | 8.361 |
| Inferior Frontal Gyrus_L_6_5 | 9.596 | Insular Gyrus_L_6_5 | 6.967 | Superior Temporal Gyrus_R_6_2 | 7.668 |
| Hippocampus_L_2_1 | 9.078 | Inferior Frontal Gyrus_L_6_1 | 5.990 | Posterior Superior Temporal Sulcus_L_2_2 | 6.386 |
| Middle Frontal Gyrus_L_7_1 | 8.146 | Insular Gyrus_L_6_4 | 5.165 | Inferior Temporal Gyrus_R_7_5 | 5.863 |
| Inferior Frontal Gyrus_R_6_5 | 8.089 | Inferior Temporal Gyrus_R_7_5 | 5.110 | Sfg_L_7_7 | 5.775 |
| Parahippocampal Gyrus_L_6_4 | 7.637 | Inferior Parietal Lobule_R_6_5 | 5.092 | Middle Frontal Gyrus_L_7_4 | 5.186 |
| Middle Temporal Gyrus_R_4_1 | 6.962 | Middle Frontal Gyrus_L_7_4 | 5.061 | Middle Frontal Gyrus_L_7_1 | 4.957 |
| Superior Temporal Gyrus_R_6_2 | 5.972 | Middle Temporal Gyrus_L_4_4 | 4.797 | Occipital Gyrus_L_4_2 | 4.935 |
| Superior Occipital Gyrus_R_2_2 | 5.914 | Cuneus_R_5_2 | 4.581 | Striatum_L_6_3 | 4.906 |
| Cingulate Gyrus_R_7_1 | 5.696 | Orbital Gyrus_L_6_6 | 4.544 | Thalamus_L_8_6 | 4.788 |
| Middle Temporal Gyrus_L_4_4 | 5.125 | Superior Temporal Gyrus_R_6_3 | 4.477 | Cuneus_R_5_3 | 4.663 |
| Precuneus_R_4_2 | 4.866 | Inferior Frontal Gyrus_R_6_5 | 4.298 | Orbital Gyrus_R_6_6 | 4.596 |
| Cingulate Gyrus_R_7_7 | 4.694 | Inferior Temporal Gyrus_L_7_6 | 4.298 | Fusiform Gyrus_L_3_1 | 4.596 |
| Middle Frontal Gyrus_R_7_4 | 4.615 | Cingulate Gyrus_R_7_1 | 4.249 | Superior Temporal Gyrus_L_6_3 | 3.521 |
| Superior Temporal Gyrus_L_6_3 | 4.522 | Precuneus_L_4_4 | 3.819 | Inferior Parietal Lobule_R_6_5 | 3.521 |
| Middle Frontal Gyrus_L_7_4 | 4.443 | Fusiform Gyrus_L_3_1 | 3.677 | Precuneus_R_4_2 | 3.337 |
| Inferior Parietal Lobule_R_6_1 | 4.263 | Inferior Frontal Gyrus_R_6_6 | 2.616 | Insular Gyrus_R_6_3 | 3.241 |
| Precuneus_L_4_4 | 4.213 | Middle Frontal Gyrus_L_7_5 | 2.462 | Inferior Frontal Gyrus_R_6_2 | 3.086 |
| Thalamus_L_8_6 | 3.811 | Superior Temporal Gyrus_R_6_2 | 2.364 | Fusiform Gyrus_R_3_1 | 3.086 |
| Occipital Gyrus_L_4_1 | 3.474 |  |  | Occipital Gyrus_L_4_1 | 3.079 |
| Middle Temporal Gyrus_L_4_1 | 3.338 |  |  | Orbital Gyrus_R_6_2 | 2.079 |
| Inferior Temporal Gyrus_L_7_6 | 2.259 |  |  | Inferior Temporal Gyrus_R_7_2 | 2.079 |
| Inferior Frontal Gyrus_L_6_2 | 2.230 |  |  | Middle Frontal Gyrus_R_7_2 | 2.013 |
| Thalamus_R_8_8 | 2.137 |  |  |  |  |

**Table 3. Nodes with significant differences in BC values among the CN, MCI, and AD**

| **CN vs. MCI** | **P value** | **CN vs. AD** | **P value** | **MCI vs. AD** | **P value** |
| --- | --- | --- | --- | --- | --- |
| Parahippocampal Gyrus_L_6_4 | 0.002 | Parahippocampal Gyrus_L_6_4 | <0.001 | Parahippocampal Gyrus_L_6_4 | <0.001 |
| Precentral Gyrus_L_6_4 | 0.003 | Hippocampus_L_2_1 | 0.001 | Hippocampus_L_2_1 | 0.004 |
| Thalamus_L_8_8 | 0.005 | Precentral Gyrus_L_6_4 | 0.008 | Precentral Gyrus_L_6_4 | 0.008 |
| Parahippocampal Gyrus_R_6_2 | 0.006 | Thalamus_R_8_1 | 0.012 | Parahippocampal Gyrus_R_6_2 | 0.013 |
| Inferior Frontal Gyrus_R_6_3 | 0.009 | Parahippocampal Gyrus_R_6_2 | 0.013 | Thalamus_L_8_2 | 0.013 |
| Thalamus_L_8_2 | 0.009 | Parahippocampal Gyrus_R_6_4 | 0.013 | Cuneus_R_5_3 | 0.020 |
| Cuneus_R_5_2 | 0.010 | Thalamus_L_8_2 | 0.013 | Thalamus_R_8_1 | 0.022 |
| Insular Gyrus_L_6_5 | 0.014 | Cuneus_R_5_3 | 0.015 | Striatum_L_6_5 | 0.024 |
| Cuneus_L_5_3 | 0.016 | Parahippocampal Gyrus_R_6_5 | 0.021 | Superior Temporal Gyrus_R_6_4 | 0.030 |
| Inferior Frontal Gyrus_R_6_6 | 0.020 | Parahippocampal Gyrus_L_6_2 | 0.025 | Posterior Superior Temporal Sulcus_L_2_2 | 0.042 |
| Cuneus_R_5_4 | 0.023 | Striatum_L_6_5 | 0.025 | Superior Occipital Gyrus_R_2_1 | 0.043 |
| Superior Temporal Gyrus_L_6_3 | 0.031 | Superior Temporal Gyrus_R_6_4 | 0.029 | Inferior Frontal Gyrus_R_6_5 | 0.048 |
| Insular Gyrus_L_6_4 | 0.034 | Fusiform Gyrus_L_3_1 | 0.030 | Inferior Temporal Gyrus_R_7_7 | 0.049 |
| Middle Frontal Gyrus_L_7_3 | 0.038 | Inferior Temporal Gyrus_L_7_1 | 0.031 |  |  |
| Orbital Gyrus_L_6_6 | 0.039 | Striatum_R_6_6 | 0.037 |  |  |
| Inferior Temporal Gyrus_R_7_7 | 0.040 | Inferior Temporal Gyrus_L_7_4 | 0.041 |  |  |
| Middle Frontal Gyrus_L_7_7 | 0.043 | Insular Gyrus_L_6_6 | 0.044 |  |  |
| Parahippocampal Gyrus_L_6_2 | 0.046 | Superior Occipital Gyrus_R_2_1 | 0.047 |  |  |
|  |  | Posterior Superior Temporal Sulcus_L_2_2 | 0.048 |  |  |
|  |  | Inferior Temporal Gyrus_R_7_7 | 0.049 |  |  |
